# Assessing the discriminative ability of the respiratory exchange ratio to detect hyperlactatemia during intermediate-to-high risk abdominal surgery

**DOI:** 10.1186/s12871-022-01757-8

**Published:** 2022-07-08

**Authors:** Lydia Karam, Olivier Desebbe, Sean Coeckelenbergh, Brenton Alexander, Nicolas Colombo, Edita Laukaityte, Hung Pham, Marc Lanteri Minet, Leila Toubal, Maya Moussa, Salima Naili, Jacques Duranteau, Jean-Louis Vincent, Philippe Van der Linden, Alexandre Joosten

**Affiliations:** 1 Department of Anesthesiology and Intensive Care, Université Paris-Saclay, Paul Brousse Hospital, Assistance Publique - Hôpitaux de Paris (APHP), Villejuif, France; 2 Department of Anesthesiology and Perioperative Medicine Sauvegarde Clinic, Ramsay Santé, Lyon, France; 3 Department of Anesthesiology, Paul Brousse Hospital, 12 Avenue Paul Vaillant Couturier, 94800 Villejuif, France; 4 Department of Anesthesiology, University of California San Diego, La Jolla, CA USA; 5 Department of Intensive Care, Erasme Hospital, Université Libre de Bruxelles, Brussels, Belgium; 6 Department of Anesthesiology, Brugmann Hospital, Université Libre de Bruxelles, Brussels, Belgium

**Keywords:** Tissue hypoxia, Anaerobic metabolism, Shock, Goal-directed hemodynamic therapy

## Abstract

**Background:**

A mismatch between oxygen delivery (DO_2_) and consumption (VO_2_) is associated with increased perioperative morbidity and mortality. Hyperlactatemia is often used as an early screening tool, but this non-continuous measurement requires intermittent arterial line sampling. Having a non-invasive tool to rapidly detect inadequate DO_2_ is of great clinical relevance. The respiratory exchange ratio (RER) can be easily measured in all intubated patients and has been shown to predict postoperative complications. We therefore aimed to assess the discriminative ability of the RER to detect an inadequate DO_2_ as reflected by hyperlactatemia in patients having intermediate-to-high risk abdominal surgery.

**Methods:**

This historical cohort study included all consecutive patients who underwent intermediate-to-high risk surgery from January 1st, 2014, to April 30th, 2019 except those who did not have RER and/or arterial lactate measured. Blood lactate levels were measured routinely at the beginning and end of surgery and RER was calculated at the same moment as the blood gas sampling. The present study tested the hypothesis that RER measured at the end of surgery could detect hyperlactatemia at that time. A receiver operating characteristic (ROC) curve was constructed to assess if RER calculated at the end of the surgery could detect hyperlactatemia. The chosen RER threshold corresponded to the highest value of the sum of the specificity and the sensitivity (Youden Index).

**Results:**

Among the 996 patients available in our study cohort, 941 were included and analyzed. The area under the ROC curve was 0.73 (95% CI: 0.70 to 0.76; *p* < 0.001), with a RER threshold of 0.75, allowing to discriminate a lactate > 1.5 mmol/L with a sensitivity of 87.5% and a specificity of 49.5%.

**Conclusion:**

In mechanically ventilated patients undergoing intermediate to high-risk abdominal surgery, the RER had moderate discriminative abilities to detect hyperlactatemia. Increased values should prompt clinicians to investigate for the presence of hyperlactatemia and treat any potential causes of DO_2_/VO_2_ mismatch as suggested by the subsequent presence of hyperlactatemia.

## Background

Patients undergoing abdominal surgery are at risk of an oxygen delivery (DO_2_) and oxygen consumption (VO_2_) mismatch, which can lead to postoperative complications [1]. Many studies have attempted to identify DO_2_/VO_2_ mismatch using various methods [2]. Although measurement of arterial lactate is considered standard of care for this purpose, this approach is intermittent and optimally requires an arterial catheter [3]. Although other measures, including the central venous oxygen saturation (ScvO_2_) [4], the veno-arterial CO_2_ gradient, [5] and the ratio of veno-arterial CO_2_ to arterio-venous oxygen difference [6] have shown promise, they share the same limitations as lactate measurements (i.e., intermittent and invasive blood sampling) [7].

Monitoring of the respiratory exchange ratio (RER), can reflect the presence of anaerobic metabolism in the mechanically ventilated patient [1, 8]. This ratio increases in the presence of inadequate DO_2_ for a given VO_2_ and is computed using a standard anesthesia machine gas analyzer that continuously measures inspiratory and expiratory concentrations of O_2_ and CO_2_. The RER is calculated by dividing the difference in inspiratory and expiratory CO_2_ by that of inspiratory and expiratory O_2_ values (i.e., (FeCO_2_—FiCO_2_) / (FiO_2_—FeO_2_)), and has been shown to predict postoperative complications in abdominal surgery patients. In a swine model and in abdominal surgery patients, increased RER has been associated with intraoperative hyperlactatemia [1, 9]. However, the sensitivity and specificity of the RER to detect hyperlactatemia in humans remains to be determined. If an abnormal RER can detect hyperlactatemia, it could be used as a non-invasive indicator for more aggressive hemodynamic optimization (e.g., fluid, vasopressors, and blood administration) and additional monitoring, especially as this variable can be easily calculated at the bedside during surgery. The present study tested the hypothesis that RER calculated at the end of surgery could help detect hyperlactatemia in patients undergoing intermediate-to-high risk abdominal surgery.

## Methods

The Academic Erasme University Hospital (Université Libre de Bruxelles) ethical committee approved this study December 4^th^, 2020 (SRB2020_654) and waived the need for informed consent. All methods were performed in accordance with the relevant guidelines and regulations. We report this study using STROBE guidelines. Patients were identified using TrackPro (UltraGenda, Belgium), their medical records checked with MediView (IMMJ Systems, United Kingdom), and intraoperative data retrieved from the anesthetic electronic records software (Innovian, Drager, Germany). All patients from our institution aged 18 years or older were included if they:

1. Underwent elective intermediate-to-high-risk open abdominal surgery (including hepatobiliary surgery, pancreatectomy, gastrectomy, oesophagectomy, cancer debulking, cystectomy, colectomy, nephrectomy, aorto-bifemoral bypass, abdominal aortic aneurysm surgery or other major abdominal surgery requiring a laparotomy) under general anesthesia between January 1st, 2014, and April 30th, 2019. Patients who had major vascular surgery were also included if the surgery involved an abdominal incision (e.g., aortobifemoral bypass and abdominal aortic aneurysm surgery). All of these surgeries were classified as intermediate- or high-risk surgeries according to Kristensen et al. [10]
2. Had routinely arterial blood gas, arterial lactate, FiO_2,_ FiCO_2_, FeO_2_, and FeCO_2_ measurements done simultaneously at the start and end of surgery. Importantly, RER values are not displayed in real time on our intraoperative monitors but were calculated retrospectively using inhaled and exhaled O_2_ and CO_2_ values (electronic medical records).

Exclusion criteria included pregnancy, lack of arterial blood gas analysis during surgery, emergency surgery, and laparoscopy.

As arterial lactate values above 1.5 meq/L have been associated with increased mortality in multiple studies examining surgical and critically ill patients and we wanted to establish a clinical cutoff value for optimal utility by most practicing physicians, [11–13] patients were split into two groups depending on the arterial lactate value recorded at the end of surgery: above 1.5 mEq/l (i.e., high lactate) or less than or equal to 1.5meEq/l (i.e., low lactate). While splitting patients into two groups is not ideal for a continuous variable, we felt this was important for increasing clinical utility and was therefore done for exploratory purposes.

### Anaesthesia protocol

All patients had at least one large peripheral venous catheter and were monitored with standard American Society of Anesthesiology (ASA) monitoring (i.e., pulse oximetry, non-invasive blood pressure, 3 or 5 lead EKG, inhaled and expired gases, and temperature monitoring), and invasive blood pressure monitoring through a radial, femoral, or brachial artery catheter. Frontal electroencephalogram monitoring with the Bispectral index, hemodynamic pulse-contour analysis, central venous pressure, and other supplemental monitoring tools were used at the discretion of the attending anesthetist.

Anesthesia administration was not standardized. Induction agents included propofol, etomidate, and ketamine. Opioids consisted of either remifentanil or sufentanil administration. Neuromuscular blockade was administered with succinylcholine, rocuronium, or cisatracurium. Maintenance of anesthesia was done with either sevoflurane, desflurane, or propofol. Adjuvant antinociception with spinal morphine, locoregional local anesthetics (e.g., epidural, transverse abdominal plane block, etc.), and opioid sparing agents (e.g., ketamine, dexmedetomidine, dexamethasone, lidocaine, paracetamol, diclofenac) were administered at the discretion of the attending anesthetist.

Although no strict hemodynamic protocol was applied during surgical cases, anesthesiologists from our institution traditionally use vasopressors to maintain mean arterial blood pressure (MAP) above 65 mmHg. The vasopressor of choice was norepinephrine, but both phenylephrine and ephedrine were less commonly used.

### Data collection and outcomes

Patient baseline characteristics, intraoperative variables, postoperative major and minor complications, 30-day mortality, and 1-year mortality were collected. FiO_2,_ FiCO_2_, FeO_2_, and FeCO_2_ were collected within the first hour and during the last hour of surgery at a moment of ventilator stability (i.e., no modifications greater than 50 ml in tidal volume) that coincided with an arterial blood gas analysis. The primary objective was to test the hypothesis that RER measured at the end of surgery could detect hyperlactatemia at that time. As there was also an interest to explore other possible associations between patients having low vs a high lactate values, we presented these data but they should be considered purely exploratory.

### Statistical analysis

The Kolmogorov Smirnov test determined that distribution was not normal (skewness of the different variables) and continuous variables were thus reported as median with quartiles [25^th^ -75^th^ percentile]. Discrete variables were reported as frequencies (with proportions). Our primary analysis was the estimation of the area under the receiver operating characteristics (AUROC) curve to establish discriminative properties of the RER to detect hyperlactatemia. We first fit a logistic regression model to estimate the ROC curve. We then estimate the AUROC according to the Delong et al. methodology and its 95% confidence intervals with the calculation of an exact Binomial Confidence Interval [14].  From the ROC curves, the optimal cut-off value yielding the greatest combined sensitivity and specificity was measured. We defined values within the 95% CI of the obtained threshold value as an inconclusive (gray zone) according to Cannesson et al. [15]. This gray zone approach is a zone of uncertainty which explores the clinical usefulness of the RER to detect hyperlactatemia. Statistical analysis was conducted with MedCalc® Statistical Software version 19.6.4 (MedCalc Software Ltd, Ostend, Belgium; https://www.medcalc.org; 2021).

**Fig. 1:**
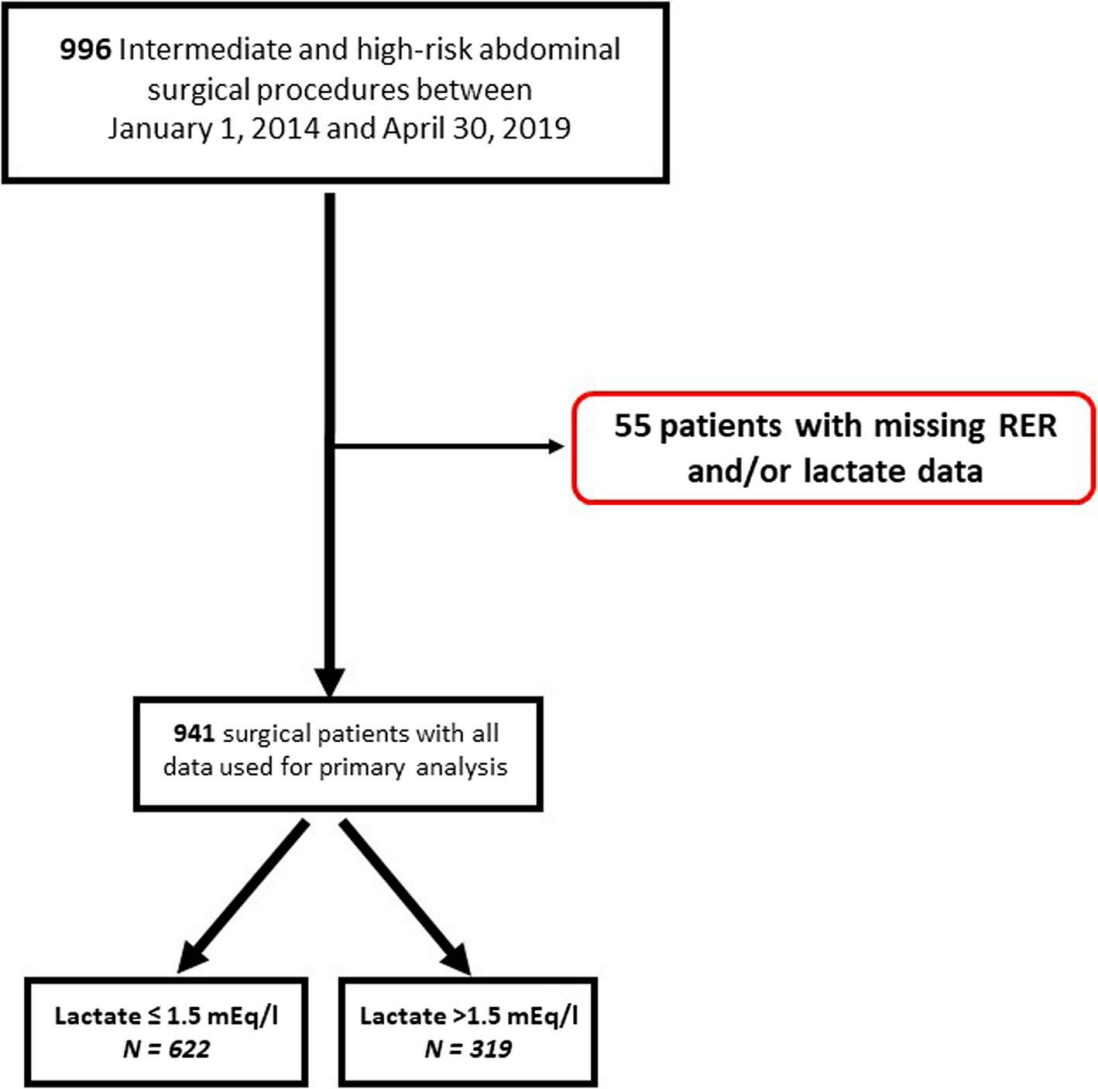
Flow Chart

A total of 996 patients undergoing intermediate- to-high risk abdominal surgery were eligible, of whom 55 were excluded due to missing data. Consequently, 941 patients were included, 622 being in the low lactate group and 319 in the high lactate group (Fig. 1).

**Fig. 2:**
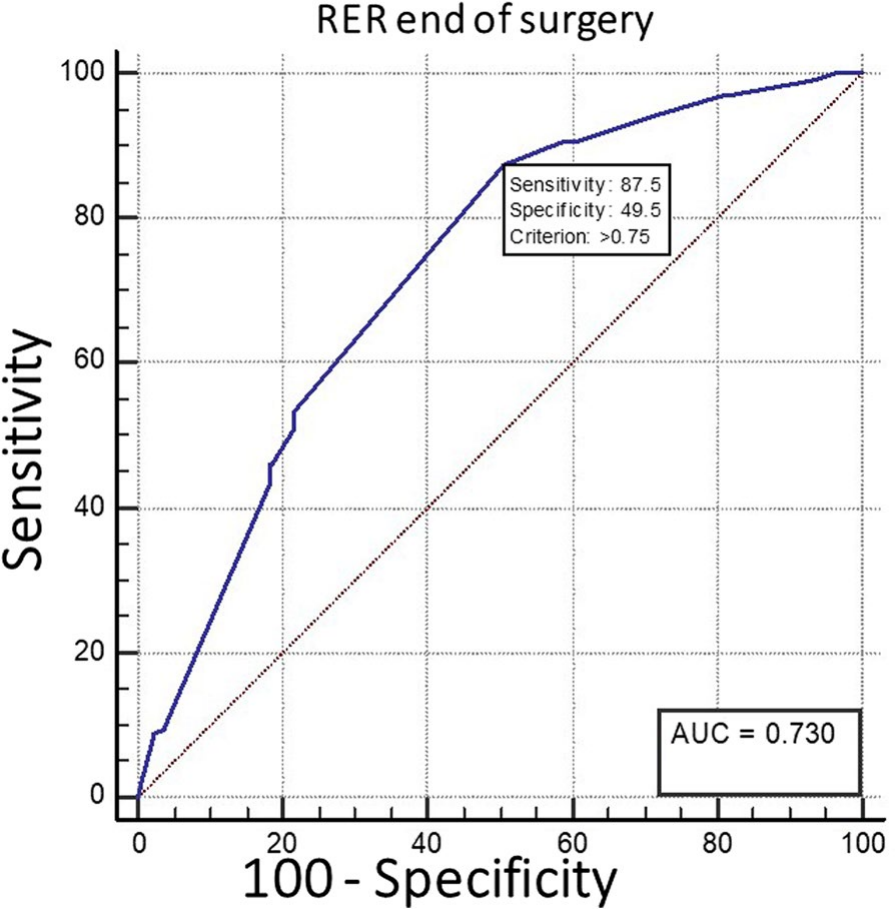
Receiver operating characteristic curve to examine if the RER at the end of the surgery can detect hyperlactatemia

A RER of > 0.75 (Youden index) at the end of surgery detected a lactate value above 1.5 mEq/L with a sensitivity of 87.5% and a specificity of 49.5%. The area under the receiver operating characteristic curve was 0.730 (95% CI: 0.70 to 0.76; *p* < 0.001) (Fig. 2). Using a sensitivity of 90% and a specificity of 90%, four hundred and seven patients (43%) were in the grey zone defined from a RER of 0.72 to a RER of 0.98 (283 patients had a RER < 0.72 and 251 patients a RER > 0.98, thus having respectively a false negative rate and a false positive rate of 10% or below (relative high certainty)).

RER was significantly higher in the high lactate group at the beginning and at the end of surgery (Table 2). Overall, baseline characteristics were not different between groups except for body mass index, which was higher in the high lactate group; preoperative hemoglobin, which was lower in the high lactate group; preoperative aspirin use, which was more frequent in the low lactate group; and the type of surgery (Table 1). Intraoperative variables were significantly different between the two groups with respect to anesthesia and surgery times, fluids infused, blood products administered, net fluid balance, vasopressors, lactate, and RER, indicating more challenging intraoperative conditions in the high lactate group (Table 2). While these differences may indicate potential confounding factors, our intention was simply to determine if postoperative RER values could be useful to help detect increased arterial lactate levels, irrespective of differences in patients’ baseline clinical characteristics.

Exploratory secondary objectives were not different between groups (Table 3).

**Table 1:** Baseline characteristics

Variables	Lactate ≤ 1.5 mEq/l (*N* = 622)	Lactate > 1.5 mEq/l (*N *= 319)	*P*-value
Age (years)	66 [56–73]	64 [55—72]	0.078
Sex, Female (%)	220 (35.4%)	125 (39.2%)	0.258
BMI (kg/m^2^)	25.17 [22.5–28.86]	25.8 [23.31–29.4]	0.031
ASA score (1–2 / 3–4)	361 / 261	193 / 126	0.125
Preoperative hemoglobin (g/dL)	13.1 [11.8–14.3]	13.5 [12.1–14.5]	0.013
Preoperative creatinine (mg/dL)	0.9 [0.7–1.1]	0.9 [0.71–1]	0.850
***Comorbidities; N (%)***			
Ischemic heart disease Coronary artery bypass graft Hypertension Hyperlipidemia Stroke Atrial fibrillation Heart failure Diabetes mellitus 1 Diabetes mellitus 2 COPD Cirrhosis Asthma	63 (10.1%) 34 (5.5%) 319 (51.3%) 179 (28.8%) 25 (4.0%) 49 (7.9%) 10 (1.6%) 2 (0.3%) 138 (22.2%) 84 (13.5%) 43 (6.9%) 30 (4.8%)	24 (7.5%) 11(3.5%) 144 (45.1%) 86 (27.0%) 15 (4.7%) 20 (6.3%) 4 (1.3%) 2 (0.6%) 66 (20.7%) 30 (9.4%) 30 (9.4%) 19 (6.0%)	0.192 0.170 0.074 0.557 0.623 0.370 0.669 0.495 0.598 0.068 0.176 0.459
***Medications; N (%)***			
Aspirin Clopidogrel ẞ blocker ACEI ARB Calcium channel blocker Diuretics Statin Oral hypoglycaemic drugs Insulin Oral anticoagulation	224 (36.0%) 30 (4.8%) 172 (27.7%) 136 (21.9%) 50 (8.0%) 119 (19.1%) 65 (10.5%) 181 (29.1%) 94 (15.1%) 49 (7.9%) 64 (10.3%)	85 (26.7%) 8 (2.51%) 81 (25.4%) 57 (17.9%) 22 (6.9%) 49 (15.4%) 25 (7.8%) 91 (28.5%) 47 (14.7%) 27 (8.5%) 26 (8.2%)	0.004 0.088 0.459 0.151 0.533 0.153 0.197 0.854 0.877 0.755 0.291
***Type of Surgery (N)***			< 0.001
Pancreatectomy Hepatobiliary Oesophagectomy Cystectomy Cancer debulking Major vascular surgery Gastrectomy Colectomy Nephrectomy Other surgical procedure^a^	104 (16.7%) 161 (25.9%) 82 (13.2%) 72 (11.6%) 25 (4.0%) 141 (22.7%) 7 (1.1%) 17 (2.7%) 7 (1.1%) 6 (0.1%)	58 (18.2%) 152 (47.7%) 37 (11.6%) 18 (5.6%) 10 (3.1%) 32 (10.0%) 1 (0.3%) 6 (1.9%) 2 (0.6%) 3 (0.9%)	

Values are presented as medians [interquartiles ranges] or numbers (percentages %)

^a^Included other laparotomies such as surrenalectomy or prostatectomy

**Table 2:** Intraoperative Variables

Variables	Lactate ≤ 1.5 mEq/l (*N *= 622)	Lactate > 1.5 mEq/l (*N* = 319)	*P*-value
Anaesthesia duration ( min)	347 [254–450]	372 [287–472]	0.001
Surgery duration (min)	262 [180–360]	289 [209–381]	0.002
Total crystalloid (ml)	2000 [1200–3000]	2400 [1500–3500]	< 0.001
Total colloid (ml)^b^	500 [500–1000]	1000 [500–1500]	< 0.001
Total blood product (ml)	498 [261–580]	540 [288–1475]	0.009
Total IN (ml)	2500 [1500–3500]	3000 [2000–4500]	< 0.001
Estimated blood loss (ml)	400 [200–900]	700 [300–1500]	< 0.001
Diuresis (ml)	300 [150–500]	300 [150–510]	0.258
Total out (ml)	870 [500–1400]	1110 [680–2000]	< 0.001
Net fluid balance (ml)	1505 [788–2350]	1750 [1000–2800]	< 0.001
Use of vasopressors, N (%)^a^	484 (77.9%)	269 (84.3%)	0.02
Lactate beginning of surgery (mEq/L)	0.7 [0.6–0.9]	0.9 [0.7–1.2]	< 0.001
RER beginning of surgery	0.80 [0.67–0.80]	0.80 [0.71–0.83]	< 0.001
Lactate end of surgery (mEq/L)	0.9 [0.7–1.1]	2.3 [1.8–3.0]	< 0.001
RER end of surgery	0.80 [0.67–0.80]	0.83 [0.80–1.0]	< 0.001

Values are presented as medians [interquartiles ranges] or numbers (percentages %)

^a^use of any vasopressor (ephedrine, phenylephrine, noradrenaline)

^b^total colloid included 3% gelatin and 6% tetrastarch

**Table 3:** Postoperative outcome

Variables	Lactate ≤ 1.5 mEq/l (*N* = 622)	Lactate > 1.5 mEq/l *(N* = 319)	*P*-value
**Secondary outcomes**			
**1)** **Length of stay in hospital (days)**	9 [6—14]	9 [9—28]	0.426
**2) ** **Minor complications; N (%)**	147 (23.6%)	66 (20.7%)	0.307
➢ Superficial wound infection	22 (3.5%)	7 (2.2%)	0.259
➢ Urinary infection	37 (5.9%)	13 (4.1%)	0.225
➢ Paralytic ileus	22 (3.5%)	12 (3.8%)	0.861
➢ Pneumonia	19 (3.1%)	8 (2.5%)	0.634
➢ Postoperative confusion	23 (3.7%)	10 (3.1%)	0.657
➢ Other infection	65 (10.5%)	42 (13.2%)	0.214
**3)** **Major complications; N (%)**	123 (19.8%)	72 (22.6%)	0.317
➢ Anastomotic leakage	19 (3.1%)	14 (4.4%)	0.292
➢ Peritonitis	3 (0.5%)	6 (1.9%)	0.037
➢ Sepsis	32 (5.1%)	28 (8.8%)	0.031
➢ Necrosis stoma	10 (1.6%)	2 (0.6%)	0.206
➢ Wound dehiscence	10 (1.6%)	5 (1.6%)	0.963
➢ Bleeding requiring a redo surgery	19 (3.1%)	17 (5.3%)	0.085
➢ Pulmonary embolism	5 (0.8%)	2 (0.6%)	0.765
➢ Pulmonary edema	9 (1.5%)	5 (1.6%)	0.881
➢ Acute coronary syndrome	2 (0.3%)	0 (0%)	0.678
➢ Atrial fibrillation / arrhythmia	15 (2.4%)	8 (2.5%)	0.928
➢ Acute kidney injury	34 (5.5%)	29 (9.1%)	0.035
➢ Reoperation	49 (7.9%)	17(5.3%)	0.147
➢ 30-day mortality	6 (0.1%)	4 (1.3%)	0.682

Values are presented as medians [interquartiles ranges] or numbers (percentages %)

In mechanically ventilated patients undergoing intermediate to high-risk abdominal surgery, the RER had moderate discriminative properties to detect hyperlactatemia. Based on a grey zone approach, 43% of the patients lied in an uncertainty zone with limited clinical usefulness. However, a RER value above 0.75 can detect hyperlactatemia with a relatively high sensitivity (88%). Hence, a normal RER makes hyperlactatemia relatively unlikely. Since its specificity is rather poor (50%) at this level, an increased RER cannot definitely rule in the presence of hyperlactatemia.

Bar and colleagues recently demonstrated the potential of RER to predict postoperative complications following both open abdominal high-risk and laparoscopic surgeries [1, 8]. During high-risk open abdominal surgery, both lactate and RER were found to predict postoperative complications, with an AUC of 0.77 and 0.67, respectively [1]. This confirms the importance of these measurements. Although this team did demonstrate an association between these two measurements, the sensitivity of specificity of RER to detect hyperlactatemia was not investigated [1].

In our study, patients in the high lactate group had more challenging intraoperative conditions as surgery time, fluid requirements, vasopressor infusions, and blood loss were all greater than in the low lactate group. The major postoperative complications of sepsis, peritonitis, and renal injury confirm that adverse postoperative outcomes are associated with hyperlactatemia. Other variables have been related to tissue hypoperfusion, such as ScvO_2_, the veno-arterial CO_2_ gradient, the ratio of veno-arterial CO_2_ to arterio-venous oxygen. Unfortunately, these measures, similar to arterial lactate, require access to arterial, central venous, or even pulmonary artery blood sampling. RER has the advantage of being easy to calculate, non-invasive, free and its components continuously displayed on all modern anesthesia machines. It is easy to imagine a clinical scenario in which an elevated RER following surgery results in changes in post-operative clinical management. For example, the patient with an elevated RER may require a higher level of care, additional monitoring, supplementary laboratory tests or any other treatment that can correct DO_2_/VO_2_ mismatch. Such interventions could include a fluid challenge, administration of vasoactive agents to increase cardiac output or to target a higher blood pressure, or a red blood cell transfusion to increase the hemoglobin level. Conversely, a normal RER value could potentially accelerate the postoperative care and limit unnecessary tests and excessive length of stay in a high dependency unit. In addition, in patients equipped with an arterial line, RER calculation could justify less frequent arterial blood gas measurements to check lactate levels. Further clinical exploration and the eventual implementation into goal-directed protocols may help further clarify the DO_2_/VO_2_ relationship and establish the best clinical use for RER. Additional data should be soon available as a French team completed recently a large randomized controlled trial (N = 350) comparing an individualized hemodynamic optimization strategy guided by indirect measurement of the RER to a routine care in major surgery [16]. 

This retrospective study had both strengths and limitations. It was not possible to couple lactate and RER measurements more frequently intraoperatively due to the lack of a protocolized approach to sampling blood. Consequently, only the first and last arterial lactate values were compared to concomitant RER values. Patients without any arterial blood gas values were excluded which represent 5.5% of the study collective. Imputation methods for missing data were not performed as it is never recommended to impute a missing outcome since it would only improve predictive properties. Moreover, missingness around 5% is usually considered as only creating limited bias [17, 18]. Anesthesia practice was not standardized, but this reflects typical clinical practice. Likewise, the population heterogeneity reflects real life practices. Types of surgeries (e.g., one-lung ventilation or hepatic resection) and patient comorbidities (e.g., chronic obstructive pulmonary disease), could have effects on either DO_2_ or lactate metabolism and may alter the relation between RER and lactate. Future studies should investigate the impact of these conditions on the relationship between RER and hyperlactatemia.

In conclusion, the RER had moderate discriminative abilities to detect hyperlactatemia. A RER value above 0.75 can detect hyperlactatemia with a moderately high sensitivity but with a poor specificity. Increased values should prompt clinicians to investigate for the presence of hyperlactatemia and treat any potential causes of DO2/VO2 mismatch as suggested by the subsequent presence of hyperlactatemia.

## Data Availability

The database is closed and there is no public access. However, permission to access and use the database can be obtained if necessary by request to the corresponding author.

## Abbreviations

RER: Respiratory exchange ratio
DO_2_: Oxygen delivery
VO_2_: Oxygen consumption
ScvO_2_: Central venous oxygen saturation
AUC: Area under curve
ROC: Receiver operating characteristics
